# Special Issue: Advanced Materials in Drug Release and Drug Delivery Systems

**DOI:** 10.3390/ma14041042

**Published:** 2021-02-23

**Authors:** Katarzyna Winnicka

**Affiliations:** Department of Pharmaceutical Technology, Faculty of Pharmacy, Medical University of Bialystok, Kilinskiego 1, 15-089 Bialystok, Poland; kwin@umb.edu.pl; Tel.: +48-85-7485615

Development of new drug molecules is costly and requires longitudinal, wide-ranging studies; therefore, designing advanced pharmaceutical formulations for existing and well-known drugs seems to be an attractive device for the pharmaceutical industry. Properly formulated drug delivery systems can improve pharmacological activity, efficacy and safety of the active substances. Advanced materials applied as pharmaceutical excipients in designing drug delivery systems can help solve problems concerning the required drug release—with the defined dissolution rate and at the determined site. Novel drug carriers enable more effective drug delivery, with improved safety and with fewer side effects. Investigations concerning advanced materials represent a rapidly growing research field in material/polymer science, chemical engineering and pharmaceutical technology. Exploring novel materials or modifying and combining existing ones is now a crucial trend in pharmaceutical technology.

The Special Issue “Advanced Materials in Drug Release and Drug Delivery Systems” was established to present the most recent insights into utilization of different materials with promising potential in drug delivery and into different formulation approaches that can be used in the design of pharmaceutical formulations. The brief summary of eleven articles included in the issue is presented below.

The papers [1,2,3] present development in the biomedical field using nano- or microparticulate carriers. Wang et al. provide information about developing an effective and relatively simple one-pot technique to load graphene quantum dots (GQDs) into nanoparticles intended for fluorescent imaging offering a new way to visualize the distribution and metabolism of nanoparticles in vivo without radioactive damage. It was shown that designed GQD-loaded optical magneto ferroferric oxide@polypyrrole (Fe_3_O_4_@PPy) core-shell nanoparticles are characterized by high potential applicability in monitoring the distribution and metabolism of nanoparticles and in long-term in vivo real-time tracking [1]. Zheng et al. [2] describe information about the preparation and characterization of methoxy poly(ethylene glycol)-poly(lactide) copolymer (mPEG-PLA) microspheres as carriers of alkaloids from *Alstonia scholaris* leaves—a traditional Chinese drug used in the treatment of chronic respiratory diseases. The limiting factor connecting administration of these alkaloids is short half-life, so the authors focused on the development of a multicompartment dosage form providing sustained drug release. mPEG-PLA microspheres with alkaloids from *Alstonia scholaris* leaves were obtained by w/o/w double emulsion method. Designed particles significantly prolonged drug release, were not cytotoxic in HL-7702 cells, and inhibited auricle and pedal swelling evaluated in mice and rats. Wasilewska et al. [3] report data on the exploitation of ethylcellulose microparticles containing rupatadine fumarate in the development of orodispersible minitablets—a novel solid dosage form connecting benefits of both liquid and solid formulations. As rupatadine fumarate—a second generation antihistamine drug—is characterized by bitter taste, an attempt was made to formulate “patient friendly” orodispersible minitablets with acceptable taste. To mask rupatadine fumarate’s bitterness, the drug was enclosed in microparticles obtained with widely used in the pharmaceutical technology, non soluble in water ethylcellulose. In this work, orodispersible minitablets containing rupatadine fumarate were formulated by direct compression of commercially ready to use blends and microparticles prepared with an aqueous ethylcellulose dispersion. Developed formulations of minitablets possessed beneficial pharmaceutical parameters and provided a taste-masking effect evaluated by three alternative techniques (e-tongue, human taste panel, and the in vitro drug release). It was indicated that ethylcellulose microparticles can be successfully used in the direct compression. A more detailed description of ethylcellulose applicability in drug dosage forms development is presented in the review paper by Wasilewska et al. [4]. Ethylcellulose is a multifunctional, hydrophobic cellulose derivative possessing advantageous and unique properties to be used in pharmaceutical formulations. This water insoluble polymer is regarded as safe, biocompatible, is degraded into nontoxic products, and shows gastroresistance. Ethylcellulose is widely utilized in the pharmaceutical industry, it is a valuable excipient to modify drug release profiles and to create controlled release dosage forms. In this article, particular attention was placed on its role in the oral and topical formulations.

The papers [5,6] present data about multilayer films and electrospun matrices as advanced materials used in drug delivery systems. Nie et al. [5] reported on the versatile layer by layer highly stable multilayer films prepared from polymer (hyaluronic acid) and inorganic–mesoporous silica nanoparticles for the delivery of FITC-labeled short peptides. The described method improves the stability of the multilayer structure by forming covalently cross-linked super strata on the outlying areas of the films. Moreover, the super stratum functions as a “nano net” reducing nanoparticles diffusion and is simultaneously permeable for small molecules, which can be used to develop multifunctional drug delivery systems based on mesoporous silica as the active substance depot. Mesoporous silica nanoparticles enhance the stability of peptides, retention properties of peptides can be modified by varying capping-layer numbers, and the release profile of small molecules can also be adjusted by adequate carrier arrangements. Nazarkina et al. describe sirolimus matrices for coating vascular stents [6]. Development of stent coatings is a valid issue, as stent implantation is a frequently performed medical procedure which can initialize various reactions stimulating an inflammatory response. In this work, an attempt was made to design matrices with sirolimus-drug possessing immunosuppressive and antiproliferative activity and approved both in Europe and United States as stent-coating component. Matrices were formulated by electrospinning polycaprolactone, human albumin, hexafluoroisopropanol, and dimethyl sulfoxide, and the optimal composition, the most applicable for bare-metal stent coating, ensuring prolonged sirolimus release to provide its concentration preferable for antiproliferative activity, was indicated.

The next three papers are devoted to the ever-expanding technological approaches in solid oral dosage forms like tablets [7,8] and capsules [9]. Jamróz et al. focus on the deep analysis of the relationship between structure and pharmaceutical characteristics in 3D printed tablets. Application of 3D printing in pharmaceutical technology is relatively novel, requires proper selection of excipients, and to date, only one medicinal product obtained by this technique is commercially available (Spritam, by Aprecia Pharmaceuticals, registered in the US). In this work, the composition of the 3D printed tablets and the relationship between the tablet geometry, tablet shape, and drug release profile was discussed. To evaluate the influence of a degree of infill on the printing reproducibility and to analyze the tablet structure, micro-computed tomography was used. Authors developed 3D printed immediate release tablets with liquid crystal-forming itraconazole and indicated that optimal tablet characteristics (reproducibility during printing, optimal drug release) was assured when filament formulation of 20% itraconazole, 76% polyvinyl alcohol, and 4% crospovidone was utilized [7]. Supercritical fluid method is a relatively new technique with potential application in the pharmaceutical industry to reduce particle size and to create inclusion complexes or solid dispersions. Antosik-Rogóż et al. report on the utility of the supercritical fluid method (using supercritical CO_2_) in the development of dosage forms with poorly soluble bicalutamide-drug from the antiandrogens group used in prostate cancer treatment [8]. The influence of supercritical CO_2_ on the characteristics of binary systems with bicalutamide and polymeric excipients (Macrogol 6000, Poloxamer 407), and tablets obtained from solid dispersions was deeply described. It was shown that this process affected particle size and shape, improved the in vitro bicalutamide release profile, and it evoked changes in the drug crystallinity. The paper by Maciejewski et al. [9] provides data on the gastro resistant soft gelatin films and capsule formulation from binary-gelatin/cellulose acetate phthalate and ternary-gelatin/cellulose acetate phthalate/carrageenan films. They suggest that the discreet kinetically-limited phase separation was the crucial factor affecting the disintegration of the films. In medium, at acidic pH, gelatin undergoes swelling and dissolving processes, and exposes the cellulose acetate phthalate skeleton, which is insoluble in acidic environments. Moreover, addition of carrageenan improved this acid-resistant effect. The dissolution study performed with diclofenac sodium as a model drug from the lab-scale soft capsule formulations indicated that the proposed fillings were compatible with the films, and the drug was not released at acidic pH, but it was released at pH 6.8.

In the paper by Chyzy et al. [10], the latest scientific reports on hydrogel applications as drug dosage forms were discussed. The authors provide an in-depth description of the main formulation approaches, classification of hydrogels, their physicochemical characteristics, and their exploitation in the biomedicine and drug delivery fields (in oral, dermal, ocular, vaginal, and parenteral administration). This review also provides information on stimuli responsive hydrogels and modern approaches to improve treatment efficiency by using hydrogel formulations. Readers can achieve an interdisciplinary vision into the advances in the design, optimization, and application of hydrogel delivery systems [10]. Faizan et al., in turn, reviewed recent literature reports concerning carbon monoxide-releasing materials providing carbon monoxide (CO) for medicinal purposes [11]. At higher concentration, CO has a toxic effect, but in lower, strictly controlled doses, it can be treated as a valuable agent participating in cell signaling, with potential treatment application. The authors discuss the therapeutic properties of CO and problems connected with designing and development of effective and safe CO-releasing molecules and CO-releasing materials. They thoroughly describe the possibilities of delivering CO by using a great variety of structures and strategies available for biological research and treatment applications (e.g., copolymer assembles, micelles, nano fiber gels, nanoparticles, nanosheets, metal organic frameworks, conjugation with proteins, peptides, metallodendrimers, vitamins, nanodiamonds), and consider toxicological aspects of CO delivery involved in the development of CO-releasing materials-based pharmaceuticals.
